# Prognostic significance of multidrug-resistance protein (MDR-1) in renal clear cell carcinomas: A five year follow-up analysis

**DOI:** 10.1186/1471-2407-6-293

**Published:** 2006-12-19

**Authors:** Chiara Mignogna, Stefania Staibano, Vincenzo Altieri, Gaetano De Rosa, Giuseppe Pannone, Angela Santoro, Rosanna Zamparese, Massimino D'Armiento, Romualdo Rocchetti, Ernesto Mezza, Mario Nasti, Viviana Strazzullo, Vittorino Montanaro, Massimo Mascolo, Pantaleo Bufo

**Affiliations:** 1 Department of Biomorphological and Functional Sciences, Section of Pathology, University of Naples "Federico II", Naples, Italy; 2 Department of Surgical Sciences, Section of Urology, University of Naples "Federico II", Naples, Italy; 3 Department of Surgical Sciences, Section of Anatomic Pathology and Cytopathology University of Foggia, Foggia, Italy; 4 Department of Surgical Sciences, Section of Urology, University of Naples "S.U.N.", Naples, Italy

## Abstract

**Background:**

A large number of renal cancer patients shows poor or partial response to chemotherapy and the mechanisms have not been still understood. Multi-drug resistance is the principal mechanism by which many cancers develop resistance to chemotherapic drugs. The role of the multi-drug resistant transporter (MDR-1/P-glycoprotein), the gene product of MDR-1, and that one of the so-called multi-drug resistance associated protein (MRP), two energy-dependent efflux pumps, are commonly known to confer drug resistance.

We studied MDR-1 expression in selected cases of renal cell carcinoma (RCC), clear cell type, with long-term follow-up, in order to establish its prognostic role and its possible contribution in the choice of post-surgical therapy.

**Methods:**

MDR-1 has been studied by standard LSAB-HRP immunohistochemical technique, in paraffin embedded RCC samples. Protein expression has been compared to clinical and histopathological data and to disease specific survival of RCC patients, by Kaplan-Meier curve and Cox multivariate regression analyses.

**Results:**

Two groups of RCCs were obtained by esteeming MDR-1 expression and disease specific survival (obtained with Kaplan-Meier curve and Cox multivariate regression analyses): the first one presents low or absent MDR-1 expression and good survival; the second one is characterized by high MDR-1 expression and significant poor outcome (*p *< 0.05). Afterwards, we have found disease specific survival, adjusted for stages and independent of therapy: this difference of survival rates was statistically significant (*p *< 0.05). Stage adjusted disease specific survival rate, according to MDR-1 expression and therapy in patients affected by RCC in early stage (stage I), has revealed that the group of patients with high MDR-1 expression and without adjuvant therapy showed poor survival (*p *< 0.05). Cox multivariate regression analysis has confirmed that, in our cohort of RCC (clear cell type) patients, the strong association between MDR-1 and worse outcome is independent not only of the adjuvant therapy, but also of the other prognostic parameters (*p *< 0.05).

**Conclusion:**

In our opinion, the results of this study well prove the relationship between MDR-1 expression and worse clinical prognosis in RCC, because MDR-1 over-expressing RCCs can be considered a group of tumours with a more aggressive behavior. This finding outlines a possible role of MDR-1 as prognostic factor, dependent and independent of multidrug resistance. These results could be useful to predict cancer evolution and to choose the appropriate treatment: this is another step that can stimulate further promising and interesting investigations on broader study population.

## Background

Renal cancer is the seventh leading cause of cancer mortality, representing 2,6% of all human tumours [1]. The most frequent type of renal cell carcinoma is the conventional (clear cell) one [2]. Approximately, one third of the patients with RCC has metastatic disease at the beginning, and up to 50% relapses post-nephrectomy [3]. RCC is characterized by a poor prognosis, almost unchanged for decades, because of its late presentation and/or high degree of intrinsic or acquired resistance to chemotherapy [4].

The classical prognostic parameters, such as histological grade and type, performance status, patient age, number and site of metastases and their modality of appearance, do not always assume an unequivocal role for the correct management of RCC patients and to improve their clinical outcome. Moreover, tumour biology of RCC still remains poorly understood. So, the prognosis of the single cases of RCC often persists as unpredictable [5-10].

It is well-known that renal cancer patients often show poor or partial response to chemotherapy and the mechanism is only partially known. Multi-drug resistance, the principal mechanism by which many cancers develop resistance to chemotherapy drugs, is one of the main factors in the failure of different chemotherapy protocols. It affects patients with a variety of blood cancers and solid tumours, including breast, ovary, lung and low gastrointestinal tract cancers. Resistance to therapy has been correlated to the presence of, at least, two molecular "pumps" that actively expel chemotherapics out of tumor cells: P-glycoprotein and the multi-drug resistance associated protein (MRP) [11,12]. The multi-drug resistant transporter (MDR-1/P-glycoprotein), the gene product of MDR-1, is a glycosylated membrane protein of 170 kDa, belonging to the ATP-binding cassette superfamily of membrane transporters [12,13].

In the present study, we evaluated the role of MDR-1/P-glycoprotein expression in a selected series of 30 conventional (clear cell type) RCCs, in order to verify its value as a predictor of clinical outcome.

## Methods

### Study population

A preliminary survey was performed on an initial renal tumour population, represented by 30 RCCs (clear cell type), 3 RCCs (sarcomatoid type), 2 RCCs (cromophobe type), 1 RCC (papillary type) and 1 oncocytoma. Our starting study was carried out on all these samples, obtained from patients that underwent open-surgery at the Department of Urology of the University "Federico II", Naples, Italy, from January 1993 to December 1996. All patients have been treated with radical open-nephrectomy, including resection of peri-nephric fat, Gerota's fascia, adrenal gland and regional lymph nodes. This first research was directed to specify the most important prognostic factors in renal neoplastic pathology: DNA ploidy [14], anti and pro-apoptotic proteins (such as Bcl-2/Bcl-xl and Bax), oncosuppressors (as p53), transporter proteins (like MDR-1), thrombosis of caval vein, necrosis, multicentric pattern of growth, histotype, grading and staging. By preliminary univariate analyses of the different histopathological, immunohistochemical and clinical parameters, we could identify MDR-1 as the only immunohistochemical factor and tumour stage as the sole histopathological parameter that were characterized by values that were close to statistical significance. To standardize our study population, we selected only RCCs (clear cell type) for the further investigations and we removed the other histotypes because, in the initial sample, they represented too small numerical fractions to be studied by statistical analysis. Successively, Cox multivariate regression analysis (MVA) has been used to confirm independent predictors of outcome among histopathological, immunohistochemical and clinical variables. Therefore, 30 RCCs (clear cell type) were employed in this following study only when a complete and long-term clinical follow-up was available. The mean follow-up time of the studied cases was 69.83 months. All patients gave their informed written consent and this study has been approved by Ethical Committee of the University of Naples.

Clinical data were reviewed to record sex, age of patients and their follow-up status (Table 1).

The histopathological diagnosis of RCC was made at the Department of Biomorphological and Functional Sciences, Section of Pathology, and confirmed at the Section of Anatomic Pathology of the University of Foggia. Histological reports regard histotype, size, multicentric pattern of growth, grade and stage, necrosis, thrombosis of caval vein. Tumour extent, that, in the course of our previous surveys, was defined by Robson system, in this study, has been revised and classified according to the 2002 TNM system [15-17], for the statistical analyses. Tumour nuclear grade was defined using Fuhrman's histological classification [18].

### Immunohistochemistry

Tumour specimens for the immunohistochemical evaluation with anti-MDR-1 protein were obtained from the affected kidneys as described by Ljungberg et al. [14]. Briefly, for each formalin-fixed and paraffin-embedded sample, 4-μm serial sections were cut, dewaxed and rehydrated. After quenching endogenous peroxidase, achieving antigen retrieval and blocking non-specific binding sites, incubation with primary antibodies was carried out overnight, at room temperature, with 1:100 dilution of anti MDR-1 (polyclonal primary antibody, sc-1517, Santa Cruz Biochemistry, Santa Cruz, CA). Finally, the bounding of primary antibody was detected by the conventional LSAB-HRP procedure, using DAB as chromogen.

Serial sections, on our RCC samples, also included non-lesional areas, 5 cm distant from tumoral mass. Negative controls were performed on these sections (the non-lesional ones) and on other sections that comprised normal areas of removed kidneys for surgical non-neoplastic renal diseases; while positive control was executed on sections obtained from a case of infiltrating breast cancer. Slight nuclear counterstaining was realized with Harris' haematoxylin.

The results of the immunohistochemical staining were evaluated separately by two observers, completely unaware of the histological typing and of the follow-up data of the single cases of RCC.

Synthetically, the number of MDR-1 expressing tumour cells was estimated as a percentage of the final number of cells per section and scored in three categories: score 1 (0–20% of positive cells); score 2 (21–40% of MDR-1 expressing cells); score 3 (>40% of MDR-1 expressing cells). The intensity of staining was graded as weak (+), moderate (++), or strong (+++).

Absolute counts of immunostaining were made by scoring neoplastic cells, selected among 7–8 non-consecutive fields and chosen in the most viable areas of the lesions (at ×40 magnification) [19,20].

Inter-rate reliability between the two investigators examining the immunostained sections was assessed by the Cohen's K test, yielding K values higher than 0.70 in almost all instances.

### Statistical analysis

Data have been analyzed, utilizing GraphPad Prism version 4 and SPSS version 15 statistical softwares. Multiple observations are presented as arithmetic means with standard errors of means. Statistical evaluations were carried out using one way analysis of variance (ANOVA) and the Student-Newman-Keuls test. The probabilities of disease specific survival were calculated by Kaplan Meier estimates and Cox multivariate regression analysis. By this last multivariate statistical evaluation, first of all, we analyzed the association between tumour stage (TNM 2002 staging system) and different clinical and histopathological co-variables, controlling for sex, age, grading, tumour size, multicentric pattern of growth, thrombosis of caval vein, necrosis, adjuvant therapy and death from RCC. Finally, the same relationships have been investigated for MDR-1. Only values of *p *< 0.05 were considered as significant.

## Results

Using x-ray, bone scan, liver ultrasound, abdominal computed tomography and pathological reports, we determined TNM tumour extent of all cases, in replacement of the former Robson staging system: 15 cases in stage I, 5 in stage II, 7 in stage III and the last 3 in stage IV [21]. Furthermore, 8 patients in stage I have been submitted to adjuvant chemotherapy (Vinblastine 0.2–0.3 mg/Kg i.v.), 1 patient in stage II, 6 in stage III and nobody in stage IV.

From the histopathological examination, 6 out of 30 tumours were G1, 17 were G2, 4 were G3 and the remaining ones were G4.

During the follow-up period of at least 5 years, 19 patients remained tumour-free, 9 patients died from RCC, whereas in 2 cases, death was independent of their renal cancer.

The statistical evaluation of immunohistochemical checking has been compared with clinical reports, pathological findings and follow-up data. There was no statistically significant correlation between MDR-1 expression and sex, age, tumour size, tumour stage and histological grade (Table 2). On the other hand, we have obtained a statistically significant correlation between MDR-1 expression and follow-up status: in 84% of the tumour-free patients, the level of immunostaining displayed score 1 and, in the remaining cases, 10% corresponds to score 2 and 6% to score 3.

33% of patients who died from RCC showed score 3, 44% score 2 and only 23% of them score 1. Two patients who died from other diseases presented score 2 and 1, respectively. Therefore, patients who died because of RCC or its metastases had a higher score than patients still alive or died from other causes.

**Figure 1:**
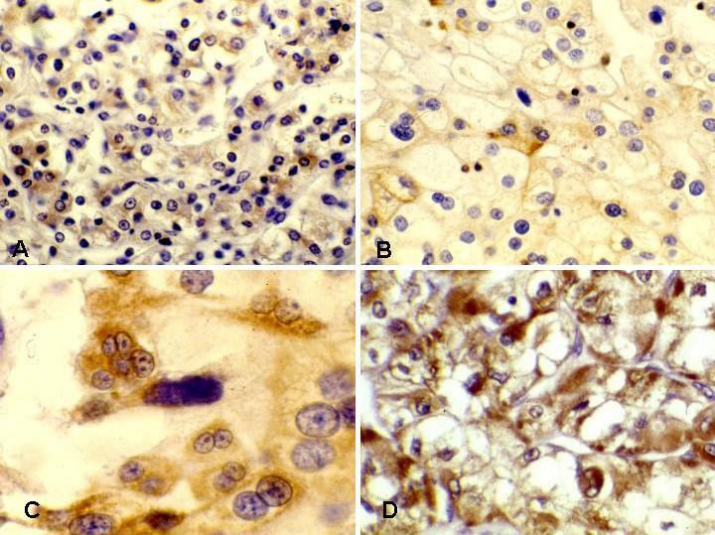
**Immunohistochemical MDR-1 expression in RCCs**. Representative examples of RCCs with low (**A: **×100; **B: **×250) and high (**C: **×400; **D: **×400) MDR-1 expression, as evaluated by immunohistochemistry (LSAB-HRP, nuclear counterstaining with haematoxylin).

In synthesis, the immunohistochemical analysis has revealed, among all the selected cases, a weak positivity in 63% of them, a moderate positivity in 23% of the patients and strong positivities in the remaining percentage of cases. Afterwards, to obtain a precise survival analysis, it has been possible to classify RCC patients in two different groups, according to the MDR-1 expression grade (Figure 1): in this way, we have found that patients with moderate-strong MDR-1 expression (score 2 – 3) had poorer survival rates, therefore a worse outcome, than the group of patients with weak or absent immunostaining (score 1).

**Figure 2:**
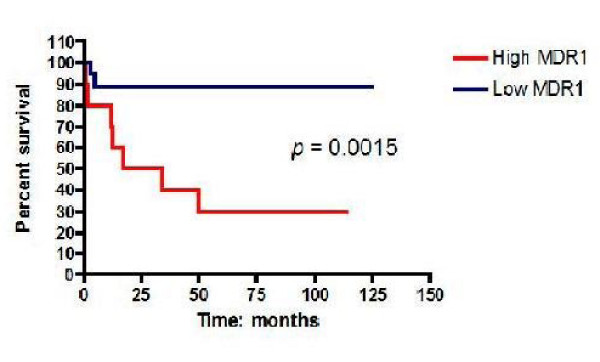
**Disease specific survival rate according to MDR-1 expression**. The high expression patients showed a significantly worse survival than low expression patients. Kaplan-Meier analysis displays a significant result (*p *< 0.05).

**Figure 3:**
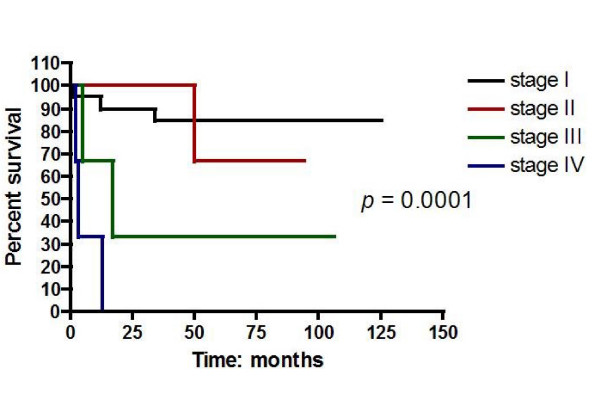
**Disease specific survival rate according to tumoral stage**. The advanced stages showed poorer survival (*p *< 0.05).

By the statistic Kaplan Meier curve (Figure 2), first of all we have analysed clinical data, referring to disease specific survival of RCC patients according to MDR-1 expression (*p *< 0.05) and, subsequently, we have found disease specific survival, adjusted for stages and independent from therapy (Figure 3). This difference of survival rates was statistically significant: patients in advanced stages had a higher mortality than patients with localized RCC (*p *< 0.05).

**Figure 4:**
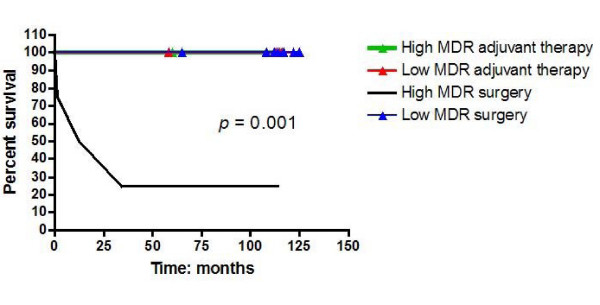
**Stage adjusted disease specific survival rate according to MDR-1 expression and therapy in patient affected by RCC in early stage (stage I)**. The group of patients with high MDR-1 expression without adjuvant therapy showed poor survival (*p *< 0.05). This finding outlines a) the role of occult metastases at the time of diagnosis and b) confirms a possible role of MDR-1 as prognostic factor independent of multidrug resistance.

Later on, to confirm the MDR-1 role as a real prognostic factor in RCCs (clear cell type), apart from a chemo-resistance marker, we excluded patients with a higher stage and selected only patients in stage I (Figure 4): the fraction of survival has statistically improved in RCCs treated with adjuvant therapy and the high expression of MDR-1 has a statistically significant correlation with a poorer prognosis, independently from using the adjuvant therapy (*p *< 0.05).

By Cox multivariate regression analysis (Table 3), we have confirmed the relationship between advanced TNM 2002 stage (stage > I) and poor prognosis (*p *< 0.05). The correlation between MDR-1 over-expression (score > 1) and high mortality rate was statistically plain and significant (*p *< 0.05), too (Table 4). Moreover, with respect to the other co-variables (sex, age, death from RCC, grading, tumour size, multicentric pattern of growth, thrombosis of caval vein, necrosis, adjuvant chemotherapy), no statistical association has been observed when tumour stage, first, and MDR-1, then, had been considered as state variables.

## Discussion

The value of classic and modern prognostic factors in renal clear cell carcinoma has been widely reported in the literature. Tumour stage is the most important independent prognostic factor. The presence or absence of distant metastases is highly prognostic and the presence of lymph node metastases is of higher prognostic value than the presence of renal vein invasion. For each given tumour stage, tumour grade (especially nuclear grade) is the most reliable additional independent prognostic factor predicting patient survival [22]. The significance of DNA ploidy as an independent prognostic factor is less clear, though it might be useful in combination with nuclear grading [23].

Patient-related potential prognostic factors such as age, sex and serologic parameters (thrombocytosis, erythrocyte sedimentation rate) are of lesser, if any, importance [24].

Data from molecular analysis on oncogenes and suppressor genes (p53), on angiogenetic factors (VEGFR), on chromosomal aberration provide additional prognostic information [25].

Considering RCC, there are still controversies about use of particular markers as prognostic factors. So, remarkable attention has been directed to the MDR-1 over-expression: it is a widely studied phenomenon in a great number of blood and solid tumours, in which it is responsible for the resistance to chemotherapy.

The relationship between MDR-1 expression and prognosis in different tumour types is variable. In any case, it has been elicited in numerous tumours [26].

MDR-1 is usually expressed in normal tissues including the liver, kidney, small and large intestines, brain, testis, muscle tissue, placenta and adrenals [27-33].

At the level of the luminal membrane of renal proximal tubules, MDR-1 accelerates drug secretion into the urine. Renal cell carcinoma derives from clonal cells lining the luminal surface of the proximal tubule, which show an intrinsic high level of expression of MDR-1 gene product. This may explain the inborn MDR-1 mRNA expression in RCC [34,35].

Although MDR-1 role in the pathogenesis of drug resistance is clear, its role as a prognostic factor in RCC is still doubtful.

In renal cell carcinoma, Duensing had already found a longer progression-free survival in patients with no or very few MDR-1 positive tumour cells compared to the group of patients with higher MDR-1 positivity [36]. However, this association was not confirmed by Oudard, who showed a tendency for a lower MDR-1 expression in advanced RCCs. From his point of view, this low level of MDR-1 expression seems independent of the duration of the disease [37].

In our study, we investigated the MDR-1 expression in all RCCs evaluated. Only the cases of patients who died from RCC showed the highest protein expression. Then, it is possible to affirm that a strong MDR-1 level suggests a poor outcome. MDR-1 seems to be an independent prognostic factor in renal clear cell carcinoma, as confirmed by Cox multivariate regression analysis.

Our results support the observation of Duensing et al.[36] of a longer progression-free survival in patients with no or very few MDR-1 positive tumour cells compared to the group of patients with several positive tumour cells, but are in disagreement with the report of Oudard et al.[37].

Hofmockel had already noted that MDR-1 expression seems to correlate with the differentiation of the RCC [38]. Our study confirms the value of MDR-1 as prognostic marker even if we have not obtained significant statistical differences of MDR-1 expression among the various histological grades.

As widely reported in literature, the importance of cancer resistance depends, at least in part, on the MDR-1 expression, up-regulated by chemotherapy. In a previous work, we had found a significant association between p53 over-expression and the presence of an aggressive RCC phenotype [39].

Many experimental evidences prove that both p53 and MDR-1 play decisive roles in chemo-resistance [13,40]. The relationship between MDR-1 and p53 is conditional, that is, dependent on the cellular environment and drug used. Mutation of p53 induces MDR-1 promoter transactivation, resulting in an increased resistance to chemotherapy and radiation [13,41-43].

By this point of view, a gene therapy, based on wild type p53, may result in a reduction of MDR-1 promoter expression and, overall, may confirm the idea of finding modulators that could be able to inhibit the MDR-1 function and, thereby, reverse multidrug resistance [13,44].

Precise determination of prognostic factors remains an essential step in the evaluation of RCC patients.

Our study, carried out, however, on a fairly small cohort of 30 RCC (clear cell type) patients with a long term follow-up, has pointed out scientific interest on the specific role of poor survival predictor that MDR-1 takes on, even if we have considered only a single tumoral histotype.

Further investigations, maybe on larger and more heterogeneous population, should address to the relationship between MDR-1 expression, renal carcinogenesis, neoplastic progression and degree of differentiation in RCC. Anyhow, our results stimulate promising hypothesis and, as for us, they may be useful not only to predict disease evolution, but also to aid oncologists in the selection of adjuvant post-surgical treatments.

We must here remember that the failure to eradicate cancer may depend on a misidentification of the target. Current therapies succeed at eliminating bulky disease but often miss on a tumour reservoir that is the source of disease recurrence and metastases. Recent advances in the understanding of tissue development cause us to revisit the process of drug resistance, to apply it to oncogenesis and tumour progression. According to the cancer stem cell hypothesis, the renal cancer-initiating cell is a transformed stem cell, which retains the essential property of self-protection through the activity of multiple drug resistance (MDR) transporters. This resting constitutively drug-resistant cell remains at low frequency among a heterogeneous tumour mass. In the context of this hypothesis, because conventional chemotherapy assures only limited efficacy, the detection of MDR-1 diffusely over-expressing RCCs may denote the need to choose patients to enroll in new therapeutic trials, increasing the therapeutic index of antineoplastic agents [35].

## Conclusion

For years, an overflowing literature has been published about MDR-1 gene expression in renal tumor cell lines at various stages of disease and treatment. However, the clinical significance of this particular protein, if it seemed obvious in the past as a factor responsible for the development of chemoresistance, is currently reconsidered. MDR-1 gene expression seems to be, at least in some instances, a hallmark of tumor cell aggressiveness, not only of chemoresistance. The failure of MDR-1 reversal trials might result from this misunderstood role of MDR-1 expression in cancer cells. In accordance to our investigations, MDR-1 renal tumoral expression and worse clinical outcome may be linked. This study has the aim to describe a new role of MDR-1 and its clinical prognostic significance in RCCs (clear cell type), suggesting that it could rather be considered as an adverse prognostic factor, however independent of therapy.

## List of abbreviations

RCC: renal cell carcinoma;

MDR-1/P-glycoprotein: multi-drug resistant transporter;

VEGFR: vascular endothelial growth factor receptor;

MRP: multi-drug resistance associated protein;

MVA: multivariate regression analysis

LSAB-HRP: linked streptavidin-biotin horseradish peroxidase;

DAB: 3,3'-diaminobenzidine.

## Competing interests

The author(s) declare that they have no competing interests.

## Authors' contributions

SS conceived of the study; DRG and BP have made substantial contribution to its design; MC, DAM, RR, MV, PG, AV, MM and ME have participated in the acquisition, analysis and clinic-pathological correlations of data; PG together with SA and ZR helped in the coordination of this work; SV, NM carried out the immunohistochemical method. Besides, the following evaluation and interpretation of the results and the statistical analysis have been performed by PG, RZ and SA. SA has been involved in drafting the manuscript and in its sequences' alignment, too; in the end, BP has revised it critically for important intellectual content and he has given final approval of the version to be published. All Authors read and approved the final manuscript.

## Pre-publication history

The pre-publication history for this paper can be accessed here:



## Figures and Tables

**Table 1:** Characteristics of study RCC (clear cell type) population. Table summarizes clinical data referring to patients' sex and age; notices about histological tumoral characteristics (such as thrombosis of caval vein, necrosis, multicentric pattern of growth and staging) and follow-up status have been reported, too.

**Age**				
Range	44–83		Mean	59.8
**Gender**				
Male	21		Female	9
**Tumour Size**				
≤ 7 cm	17		> 7 cm	13
**Thrombosis of caval vein**				
No	27		Yes	3
**Necrosis**				
No	21		Yes	9
**Multicentric pattern of growth**				
No	27		Yes	3
**TNM 2002 Staging**				
T	N0	N+	M0	M+
T1a	6	0	6	0
T1b	10	0	9	1
T2	6	1	6	1
T3a	3	0	3	0
T3b	3	0	3	0
T3c	0	0	0	0
T4	1	0	1	0
**Follow-up**				
Living patients		Died from RCC patients		Died from other causes patients
19		9		2

Table summarizes clinical data referring to patients' sex and age; notices about histological tumoral characteristics (such as thrombosis of caval vein, necrosis, multicentric pattern of growth and staging) and follow-up status have been reported, too.

**Table 2:** Statistical analysis of MDR-1 expression and associated clinic-pathological findings in RCCs. The statistical evaluation of immunohistochemical results has been compared to clinical reports, pathological findings and follow-up data. No statistically significant correlation between MDR-1 expression and sex, age, tumour size, tumour stage and histological grade has been obtained.

**Variables**	**N.**	**Score 1**	**Score 2**	**Score 3**	**MEAN ± SEM**
**Cases**	30	19	7	4	
**Age**					
< 65 years	20	12	5	3	1,55 ± 0,17
> 65 years	10	7	2	1	1,40 ± 0,22
**Sex**					
Male	21	14	4	3	1,48 ± 0,16
Female	9	5	3	1	1,56 ± 0,24
**Tumour Size**					
≤ 7 cm	17	13	2	2	1,35 ± 0,17
> 7 cm	13	6	5	2	1,69 ± 0,21
**Grading**					
G1	6	5	0	1	1,33 ± 0,33
G2	17	11	4	2	1,47 ± 0,17
G3–G4	7	3	3	1	1,71 ± 0,29
**TNM Staging**					
I (T1 N0 M0)	15	12	2	1	1,26 ± 0,15
II (T2 N0 M0)	5	2	2	1	1,8 ± 0,37
III (T1 N1 M1) (T2 N1 M0) (T3 N0 M0) (T3 N1 M0)	7	4	2	1	1,57 ± 0,29
IV (T4 N0 M0) (T4 N1 M0) (Any T Any N M+)	3	1	1	1	2,00 ± 0,57

The statistical evaluation of immunohistochemical results has been compared to clinical reports, pathological findings and follow-up data. No statistically significant correlation between MDR-1 expression and sex, age, tumour size, tumour stage and histological grade has been obtained.

SEM: standard error of mean; Score 1 (0–20% of positive cells); Score 2 (21–40% of positive cells); Score 3 (≥ 40% of positive cells).

**Table 3:** Cox multivariate regression analysis: TNM tumour staging (stage I vs stages I, II, III, IV).

**Co-variables**	**Significance (p)**
Sex	0,613
Age*	0,715
Death from disease	0,004
Grading*	0,993
Tumour Size*	0,939
Multicentric Pattern of Growth	0,181
Thrombosis of caval vein	0,075
Necrosis	0,72
Adjuvant Therapy	0,783

We have considered tumour stage as state variable in correlation with different clinical and histopathological co-variables. The only significant statistical association has been observed between tumour stage and unfavourable outcome (p = 0.004).

*: for these co-variables, we have fixed the different cut-off values (≤ 65 years vs > 65 years for Age, G1 vs G2–G3–G4 for Grading, 7 cm vs > 7 cm as regards Tumour Size).

**Table 4:** Cox multivariate regression analysis: MDR-1 expression (score > 1 vs no expression).

**Co-variables**	**Significance (p)**
Sex	0,680
Age*	0,893
Death from disease	0,016
Grading*	0,627
Tumour Size*	0,842
Multicentric Pattern of Growth	0,380
Thrombosis of caval vein	0,372
Necrosis	0,378
Adjuvant Therapy	0,336

We have considered MDR-1 as state variable in correlation with different clinical and histopathological co-variables. The only significant statistical association has been noticed between MDR-1 expression and poorer prognosis (*p *= 0.016). MDR-1 expression seems to be a prognostic factor independent of adjuvant therapy (no significant *p*).

*: for these co-variables, we have fixed the different cut-off values (≤ 65 years vs > 65 years for Age, G1 vs G2–G3–G4 for Grading, ≤ 7 cm vs > 7 cm as regards Tumour Size).
